# Characterisation of Progressive Skeletal Muscle Fibrosis in the Mdx Mouse Model of Duchenne Muscular Dystrophy: An In Vivo and In Vitro Study

**DOI:** 10.3390/ijms23158735

**Published:** 2022-08-05

**Authors:** Matteo Giovarelli, Francesca Arnaboldi, Silvia Zecchini, Laura Brigida Cornaghi, Ambra Nava, Michele Sommariva, Emilio Giuseppe Ignazio Clementi, Nicoletta Gagliano

**Affiliations:** 1 Department of Biomedical and Clinical Sciences, Università degli Studi di Milano, via G. B. Grassi 74, 20157 Milan, Italy; 2 Department of Biomedical Sciences for Health, Università degli Studi di Milano, via Mangiagalli 31, 20133 Milan, Italy; 3 Molecular Targeting Unit, Department of Research, Fondazione IRCCS Istituto Nazionale dei Tumori, via Amadeo 42, 20133 Milan, Italy; 4 Scientific Institute, IRCCS Eugenio Medea, Laboratory of Molecular Biology, via Don Luigi Monza 20, 23842 Bosisio Parini, Italy

**Keywords:** muscular dystrophy, fibrosis, collagen turnover, matrix metalloproteinases

## Abstract

Duchenne muscular dystrophy (DMD) is a rare genetic disease leading to progressive muscle wasting, respiratory failure, and cardiomyopathy. Although muscle fibrosis represents a DMD hallmark, the organisation of the extracellular matrix and the molecular changes in its turnover are still not fully understood. To define the architectural changes over time in muscle fibrosis, we used an mdx mouse model of DMD and analysed collagen and glycosaminoglycans/proteoglycans content in skeletal muscle sections at different time points during disease progression and in comparison with age-matched controls. Collagen significantly increased particularly in the diaphragm, quadriceps, and gastrocnemius in adult mdx, with fibrosis significantly correlating with muscle degeneration. We also analysed collagen turnover pathways underlying fibrosis development in cultured primary quadriceps-derived fibroblasts. Collagen secretion and matrix metalloproteinases (MMPs) remained unaffected in both young and adult mdx compared to wt fibroblasts, whereas collagen cross-linking and tissue inhibitors of MMP (TIMP) expression significantly increased. We conclude that, in the DMD model we used, fibrosis mostly affects diaphragm and quadriceps with a higher collagen cross-linking and inhibition of MMPs that contribute differently to progressive collagen accumulation during fibrotic remodelling. This study offers a comprehensive histological and molecular characterisation of DMD-associated muscle fibrosis; it may thus provide new targets for tailored therapeutic interventions.

## 1. Introduction

The extracellular matrix (ECM) accounts for up to 10% of the whole weight of skeletal muscle in humans. It plays an integrative role in muscle function and homeostasis [1,2], providing structural support to the myofibres during muscle contractions and elastic properties to the whole organ, hence linking the transmission of force from myofibres to tendons [3,4,5].

The ECM in muscles is arranged to form the endomysium, perimysium, and epimysium [6]. The endomysium surrounds each myofibre and is located in direct contact with the sarcolemma [7], playing a major role in maintaining muscle integrity and promoting myogenesis and muscle regeneration [8]. The endomysium is composed mainly of collagen type I (COL-I), type III (COL-III), and type V (COL-V) [9]. It also consists of collagen type IV (COL-IV) and laminin, forming a specialised basement domain [1,10].

The perimysium surrounds a number of myofibres delimiting a fascicle and joins with the epimysium at the muscle surface. The perimysium acts as a basic mechanical scaffold for nerves and blood vessels within the muscle tissue and allows exchanges between blood circulation and muscle cells [11,12]. The perimysium is composed mainly of COL-I and COL-III [9]. The epimysium is the thickest and strongest sheath, surrounding the entire muscle and continuing with the tendons [6]. It is composed mainly of COL-I and minor amounts of COL-III [9].

Interstitial collagen within skeletal muscle ECM represents the primary load-bearing structural protein, mainly responsible for tensile strength [2]. Collagen content and turnover are finely regulated at the level of synthesis and degradation by matrix metalloproteinases (MMPs). Collagen maturation then leads to the formation of covalent cross-links following the hydroxylation of amino acid residues by lysyl oxidase (LOX) and lysyl hydroxylase (LH) [13,14]. Collagen cross-linking thus provides the mechanical strength of collagen fibrils and fibres and is needed for their alignment.

Qualitative and quantitative modifications of collagen content and deposition may influence the ability of muscle ECM to transmit lateral forces between neighbouring fibres or from fibres to tendons [3,4,5]. In addition to its biomechanical role, the ECM niche surrounding sarcolemma is actively involved in the regulation of the muscle’s pool of satellite cells, thus influencing the muscle’s regenerative potential [15]. In this view, satellite cells are known to be sensitive to ECM modifications, including the stiffness of collagen fibres [16].

ECM is a dynamic component of skeletal muscles. Its content, structure, and turnover can be modified during physiological and pathological processes, such as physical exercise, disuse, and aging. For instance, endurance and resistance exercises can favour the turnover of ECM skeletal muscle by increasing collagen synthesis and deposition [17].

In pathological conditions, an abnormal ECM accumulation, especially of collagen, leads to skeletal muscle fibrosis, which is prominent in many chronic myopathies, eventually affecting tissue stiffness [18,19]. Fibrosis at the endomysium level may progressively enclose myofibres in a rigid coat taking up space from myofibrils and widening the capillary-to-fibre distance; this is frequently associated with a decreased elasticity and strength of muscle framework, thus limiting contraction and inhibiting the diffusion of nutrients, oxygen, and soluble factors from the bloodstream to myofibres [20,21,22].

Muscle fibrosis is a structural hallmark of Duchenne muscular dystrophy (DMD), an X-linked degenerative genetic illness caused by mutations of the DMD gene encoding the dystrophin protein. DMD is usually diagnosed in boys at 2 to 3 years of age with an incidence of 1 in 3500–5000 live male births and a prevalence of 4.8 in 100,000 males [23,24,25]. Dystrophin is an intracellular protein that anchors the ECM to the actin cytoskeleton through a protein complex, the dystrophin-associated glycoprotein complex (DGC), which spans the sarcolemma [26]. Although DMD is a multi-system disorder, the DGC disruption has devastating consequences on the skeletal muscle tissue, increasing membrane fragility and eventually fuelling muscle degeneration and necrosis [27,28]. Progressive muscle degeneration in DMD is accompanied by modifications of the ECM environment and the development of fibrosis [29].

Currently, there is no satisfactory therapy for DMD. Genetic and classical corticosteroids-based pharmacological approaches are currently being investigated in a mixed scenario where dystrophin re-expression strategies are at the forefront for gene therapy [30,31]. In this context, fibrosis has a double negative consequence for the potential treatment of DMD since it alters muscle function and stiffness properties and also reduces the amount of target muscle available for therapy and repair. Indeed, therapies that are currently being refined to decrease necrosis and trigger regeneration in muscle diseases may have little effectivity unless fibrosis is simultaneously addressed [20,21].

The spontaneous Dmd^mdx^ mutant (mdx) mouse represents the most used model for preclinical DMD research [32,33]. Mdx mouse genetically resembles human DMD and exhibits the hallmark symptoms of the disease, such as muscle weakness, respiratory insufficiency, cardiomyopathy, and histological abnormalities, although the severity of the phenotype is milder and the timescale wider than the human condition [33,34]. Despite this, in the past 30 years, the mdx mouse has been extensively used to investigate DMD pathophysiological mechanisms and to test the efficacy of therapeutic strategies, including pharmaceuticals as well as gene therapy and cell replacement approaches [35].

To date, the study of skeletal muscle fibrosis has mainly focused on the upstream events involving muscle injury and inflammation, which trigger fibroblasts to secrete collagen and other ECM components [11,36]. Relatively little is therefore known regarding collagen organisation in fibrotic muscle and how, in turn, fibrosis leads to the disruption of skeletal muscle function.

In this study, we analysed muscle fibrosis and ECM modifications as well as the underlying molecular mechanisms in young and adult mdx mice in order to characterise how fibrosis affects different muscles during the progression of the pathology. For this purpose, muscular fibrosis was assessed on histological sections from quadriceps (QD), tibialis anterior (TA), gastrocnemius (GC), and diaphragm (DF) muscles. Collagen turnover pathways were characterised in primary fibroblasts cultured from the QD muscle, one of the most involved in DMD.

## 2. Results

### 2.1. Collagen Content, Fibrosis, and Functional Correlations

**Figure 1:**
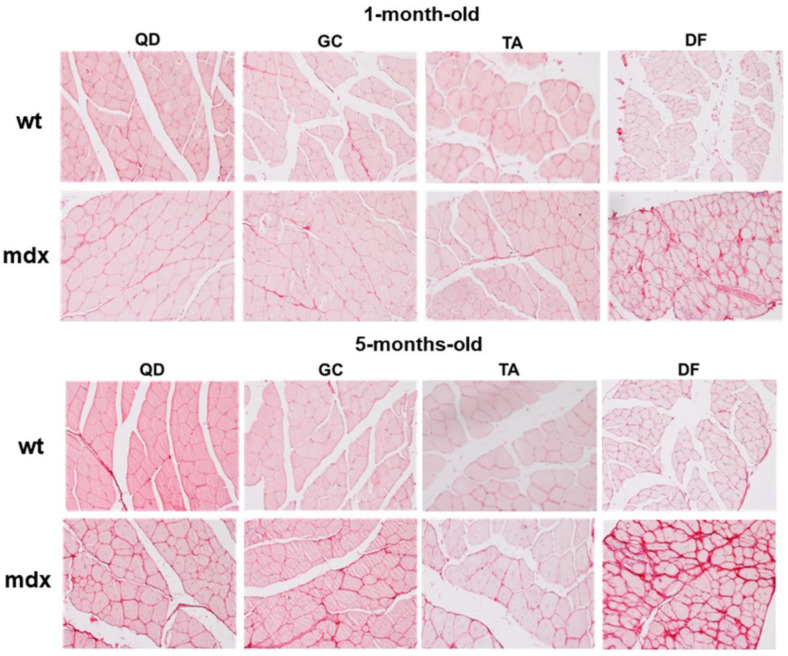
Representative Sirius red stained sections showing collagen content in quadriceps (QD), tibialis anterior (TA), gastrocnemius (GC), and diaphragm (DF) of young (1-month-old) and adult (5 months-old) mice. Original magnification: 20×.

**Figure 2:**
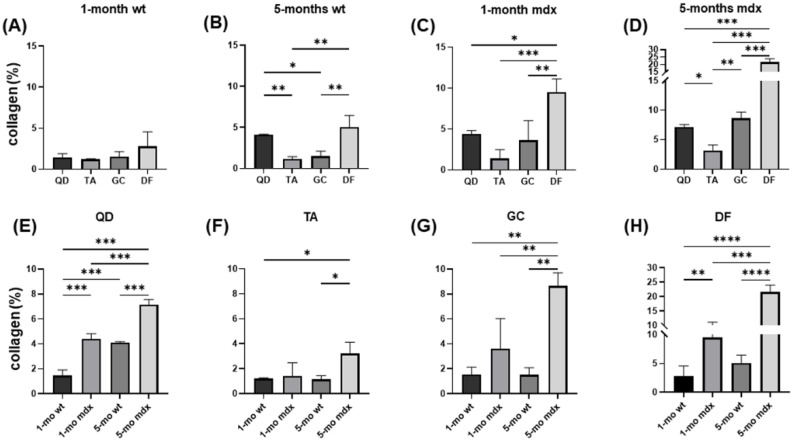
Bar graphs showing the effect of age on collagen content in sections of QD, TA, GC, DF muscles of wt and mdx mice at different age stained with Sirius red. Collagen content was assessed in the endomysium and is expressed as %. Data are mean ± SD. * *p* < 0.05; ** *p* < 0.01; *** *p* < 0.005; **** *p* < 0.001.

To obtain a comprehensive characterisation of fibrosis throughout dystrophic degeneration, we stained wt and mdx mice muscle sections with Sirius red to assess collagen content. In young wt mice, collagen content was similar in all the considered muscles (Figure 1 and Figure 2A). In adult wt mice, collagen content was significantly increased in QD and in DF (Figure 1 and Figure 2B). In young mdx mice, collagen content was significantly increased only in DF compared to the other muscles (Figure 1 and Figure 2C). Consistently with the late hypertrophic stage of muscular dystrophy at 5 months, adult mdx mice had the highest fibrosis, as shown by the strongly increased fibrosis index. Collagen accumulation in the perimysium was significant in DF. Moreover, collagen accumulated also in QD and in GC (Figure 1 and Figure 2D). These results show that DF is the most fibrotic muscle in mdx mice.

Figure 2E shows collagen content in QD. The fibrosis index is higher in young mdx compared to young wt mice as well as in adult mdx compared to adult wt mice, but to a higher extent. Moreover, QD of 5 months-old mdx mice shows a significantly higher collagen content also compared to young wt and mdx mice. In TA, the fibrosis index increased in adult mdx mice compared to adult wt (Figure 2F). GC, similar to QD, showed increased collagen content in 5-month-old mdx mice compared to the other experimental groups (Figure 2G). As previously described, DF showed the highest absolute fibrosis indexes. Indeed, collagen content was increased in young mdx vs. young wt mice, and in adult mdx mice (Figure 2H). These findings show that in both healthy and dystrophic conditions fibrosis increases with aging. They also confirm that DF is the most involved among muscles and that fibrotic muscles in mdx represent a direct marker for evaluating phenotype degeneration, performance/force impairment, as well as a direct readout of therapeutic strategies [37].

**Figure 3:**
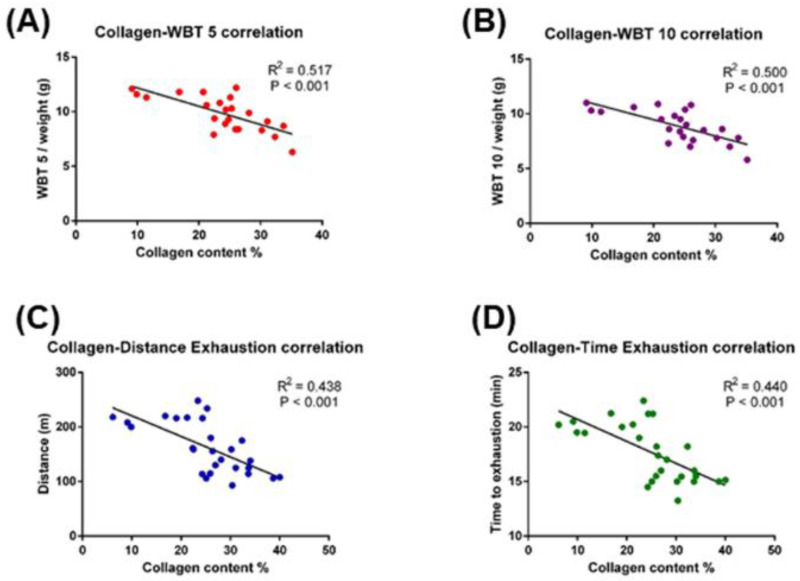
Correlation between muscle (DF) collagen content and physical performances of 5-month-old mdx mice. Collagen percentage was correlated to: WBT 5 and WBT 10 (**A**,**B**); distance ran and time to exhaustion in the treadmill exhaustion test (**C**,**D**). Pearson correlation coefficients are displayed.

Fibrosis degree directly impacts on muscles force and physical performance as shown in Figure 3. In fact, taking into consideration DF fibrosis indexes within the adult mdx mice group, muscle force scores measured by WBT inversely correlated with collagen content (Figure 3A,B). Accordingly, both treadmill exhaustion distance ran and time to exhaustion, two proxies of physical performances, significantly declined with increasing fibrosis (Figure 3C,D).

**Figure 4:**
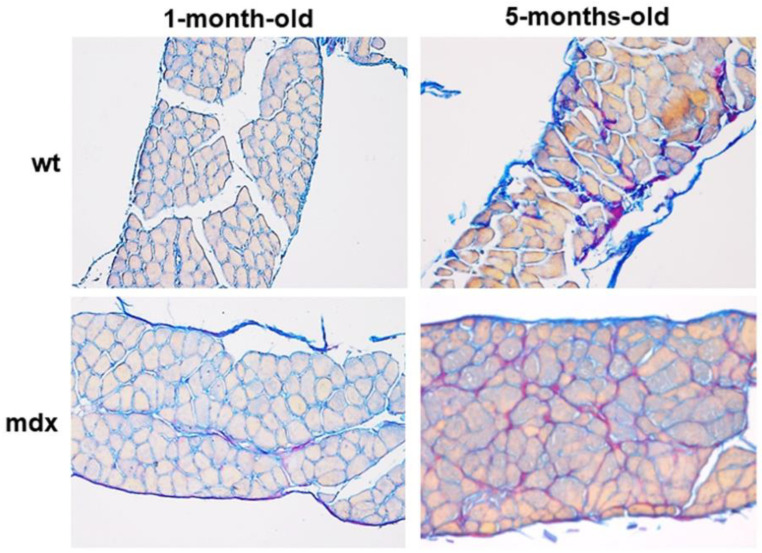
Representative sections of DF muscles stained with Herovici’s staining. The more mature and cross-linked collagen stains purple, whilst the less mature and young collagen (including reticular collagen fibres) stains blue. Original magnification: 20×.

Finally, to further characterise mdx-associated fibrosis, DF histological sections were stained with Herovici’s staining, a technique able to highlight and distinguish mature and more cross-linked collagen, which is stained purple, from the less mature and young collagen, which is stained blue. The analysis at the light microscope revealed that mature collagen in adult wt mice was more abundant than in young mice and that it was more evident in the DF of adult mdx mice (Figure 4) as well as in the other muscles (data not shown).

### 2.2. Relationship between Muscle Injury and Fibrosis

**Figure 5:**
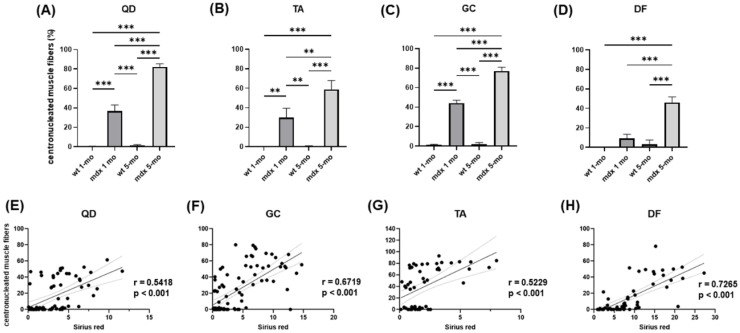
(**A**–**D**) Bar graphs showing the percentage of centronucleated muscle fibres in the considered muscles from young and adult wt and mdx mice. Data are mean ± SD. (**E**–**H**) Correlations showing the relationship between the percentage of centronucleated skeletal muscle fibres and the collagen content in the same muscles. ** *p* < 0.01; *** *p* < 0.005.

To analyse the relationship between mdx-associated muscle injury and fibrosis, we analysed the number (expressed as a percentage) of centronucleated muscle fibres, which are formed during regeneration following fibres necrosis. Centronucleated muscle fibres were almost undetectable in wt mice. Their percentage significantly increased in the QD, TA, and GC muscles of young mdx compared to wt mice, and in all the considered muscles of adult mdx compared to wt mice (Figure 5A–D). This increase was more evident in adult than in young mdx mice, suggesting that DMD induces an increased muscle regeneration in response to muscle injury.

The percentage of centronucleated skeletal muscle fibres was analysed in relation to the collagen content stained with Sirius red and assessed in the same section. The fibrosis index and the percentage of centronucleated fibres were significantly correlated in adult mdx muscles (Figure 5E–H), suggesting that the development of DMD-associated fibrosis and the extent of muscle degeneration are tightly related.

### 2.3. Non-Collagen Ground Substance Components of Muscle ECM: GAG/PG Content

**Figure 6:**
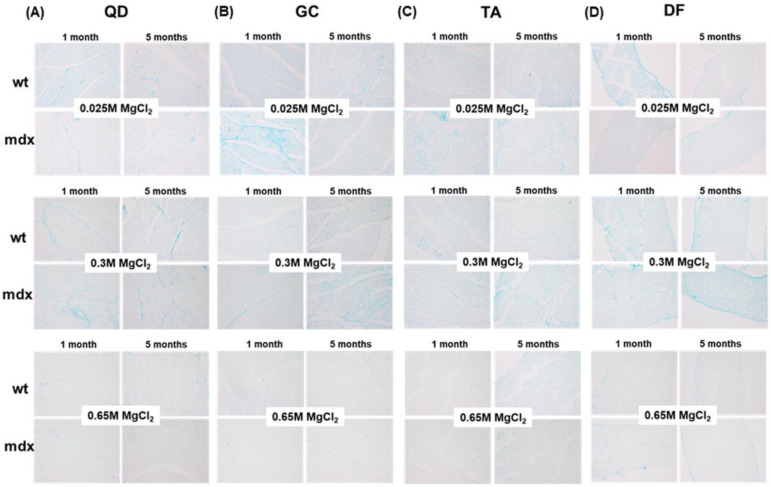
Representative micrographs of skeletal muscle sections stained with Alcian blue containing different MgCl_2_ concentrations to specifically stain and differentiate total (0.025 M), sulphated (0.3 M), or highly sulphated (0.65 M) GAG/PG content. Original magnification: 20×.

The abundance of the main components of the ground substance of ECM connective tissue, GAGs and PGs, was assessed on histological sections stained with Alcian blue. Representative micrographs are shown in Figure 6.

**Figure 7:**
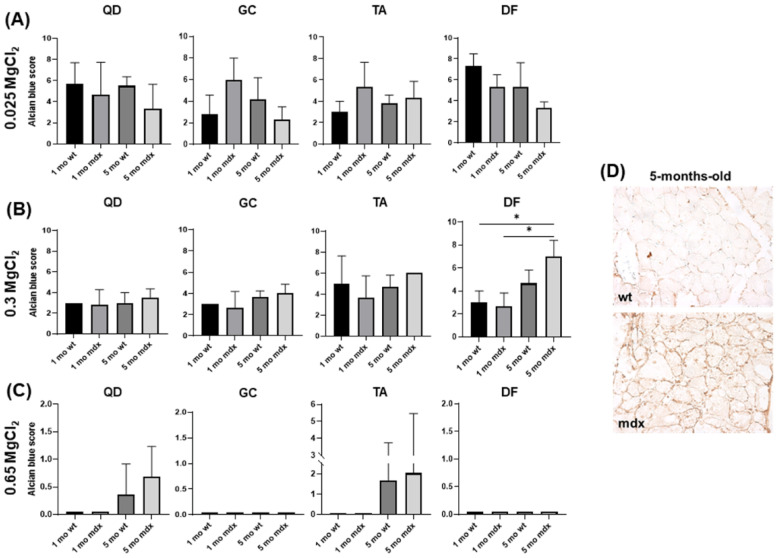
Representative bar graphs showing the score indicating GAG/PG abundance in the different muscles of young and adult wt and mdx mice after staining with Alcian blue containing 0.025 MgCl_2_ (**A**), 0.3 MgCl_2_ (**B**), and 0.65 MgCl_2_ (**C**). Data are mean ± SD. * *p* < 0.05. (**D**) Representative micrograph showing BYG immunoreactivity localisation close and around skeletal myofibres. Original magnification: 40×.

The light microscopy analysis of sections stained with Alcian blue containing 0.025 M, 0.3 M, or 0.65 M MgCl_2_ revealed a variable pattern in the considered muscles. Total GAGs/PGs stained with 0.025 M MgCl_2_ seem to slightly decrease in QD and DF of young mdx mice, while sulphated GAGs/PGs stained with 0.3 M MgCl_2_ seemed more evident in the ECM of adult mdx compared to wt mice. Highly sulphated GAGs/PGs stained with 0.65 M MgCl_2_ displayed a low signal and the sections remained pale. However, mdx sections revealed a non-homogenously distributed Alcian blue staining and localised higher expression was frequently observed. For this reason, to describe the amount of GAGs/PGs stained with Alcian blue in the different experimental groups, we expressed their content using the four-point scoring system described in the Materials and Methods Section. The quantification of Alcian blue staining using the Alcian blue score allowed for a more reliable description of GAG/PG content, as shown in the bar graphs in Figure 7.

We defined the Alcian blue score as a combination of the quantification of Alcian blue intensity and abundance. This score was not significantly affected in muscles stained in presence of 0.025 MgCl_2_, suggesting that the total GAGs/PGs content is overall not significantly modified in the different muscles of wt and mdx mice at different ages (Figure 6 and Figure 7A). Sulphated GAGs/PGs significantly increased in adult mdx DF muscle vs. young wt and mdx mice (Figure 6D and Figure 7B). Highly sulphated GAGs/PGs were observed in QD and TA muscled of adult wt and mdx mice, whereas in GC and DF they are barely detectable (Figure 6B,C and Figure 7C).

To better understand the pattern of GAG/PG expression, we analysed the expression of biglycan (BYG) by immunohistochemistry in DF. Light microscopy analysis revealed that BYG was preferentially localised around and associated with myofibres, and its expression increased in mdx compared to wt adult mice (Figure 7D).

### 2.4. Collagen Turnover Pathways in Primary Muscle Fibroblast Cultures: Collagen Synthesis and Maturation

**Figure 8:**
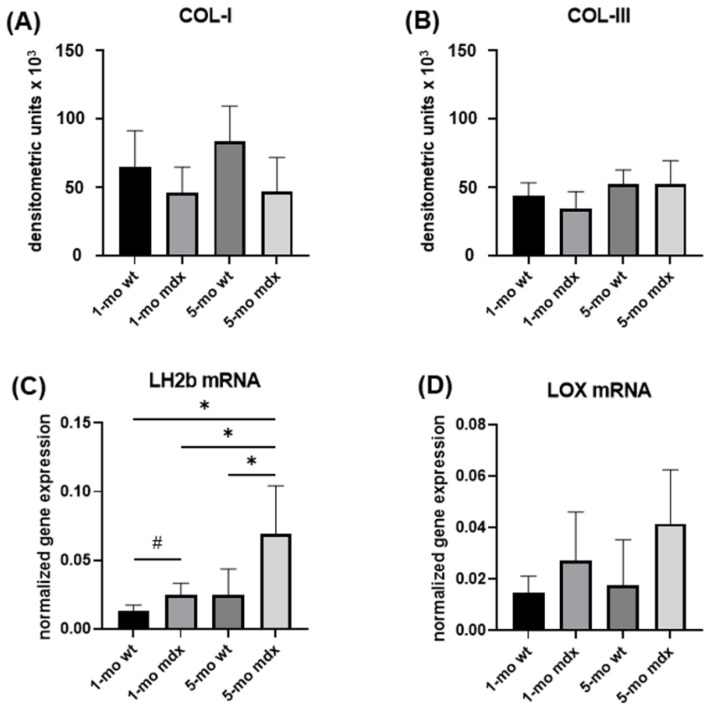
Representative bar graphs showing COL-I (**A**) and COL-III (**B**) levels in the cell culture supernatants of cultured QD muscle fibroblasts. Gene expression for LH2b (**C**) and LOX (**D**) were quantified by real-time PCR. Data are mean ± SD. * *p* < 0.05; ^#^ *p* < 0.05 for *t*-test.

Collagen turnover pathways were evaluated in QD fibroblasts since QD was highly fibrotic in mdx mice. Collagen synthesis and levels in the cell culture medium of muscle fibroblasts isolated from QD were assessed by Slot Blot assay. COL-I (Figure 8A) and COL-III (Figure 8B) were not significantly different in young and adult muscle fibroblasts from wt and mdx, suggesting a similar biosynthetic activity.

Collagen maturation by cross-linking was evaluated by analysing LH2b and LOX mRNA level, two key enzymes involved in collagen synthesis and maturation [13,14]. LH2b gene expression was significantly correlated with the degenerative adult mdx phenotype and was also significantly upregulated in young mdx compared with wt mice (Figure 8C). LOX mRNA levels were not significantly affected in dystrophic conditions, although an upregulation trend was evident in both young and mdx mice compared to wt (Figure 8D).

### 2.5. Collagen Turnover Pathways in Primary Cell Cultures of Muscle Fibroblasts: Collagen Degradation

Collagen degradation pathways were characterised by analysing MMP-1 and -2 levels in cell culture supernatants and the gene expression for their inhibitors TIMP-1 and 2.

**Figure 9:**
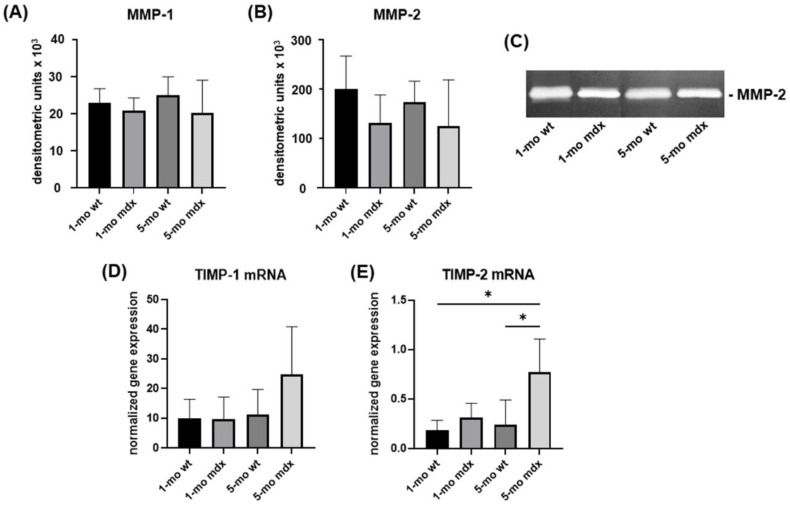
Representative bar graphs showing MMP-1 (**A**) and MMP-2 (**B**) levels in cell culture supernatants of cultured QD muscle fibroblasts analysed by Slot Blot and SDS-zymography, respectively. A representative zymogram is shown in panel (**C**). Gene expression for TIMP-1 (**D**) and TIMP-2 (**E**) were quantified by real-time PCR. Data are mean ± SD. * *p* < 0.05.

Slot Blot analysis revealed that MMP-1 levels were not significantly affected in the different experimental groups (Figure 9A). Accordingly, MMP-2 levels were not significantly affected by DMD (Figure 9B,C).

Of notice, TIMPs were upregulated in adult mdx mice. TIMP-1 gene expression was more evident in 5-month-old mdx mice, and TIMP-2 resulted significantly upregulated in 5-month-old mdx mice compared to young and adult wt mice (Figure 9D,E).

## 3. Discussion

Skeletal muscle fibres’ instability in DMD increases the susceptibility to mechanical stress during muscle contraction; this leads to progressive myofibres damage, deleterious calcium influx, release of pro-inflammatory cytokines, and the onset of mitochondrial dysfunction, eventually compromising both skeletal and cardiac muscles [37,38]. In this backdrop, muscle regeneration is sustained to counteract myofibres’ necrosis and loss; this induces the exhaustion of the satellite cells pool over time, leading to altered healing processes that result in fibrosis and fatty tissue infiltrates [27,39]. Of notice, the absence of dystrophin can directly influence the ECM homeostasis by either allowing leakage of muscle fibres components to the endomysial space or by aberrant cellular uptake of trophic factors. On the other hand, the crosstalk between interstitial muscle cell populations (i.e., resident fibroblast, fibro-adipogenic precursors, and immune cells) within the dystrophic muscle milieu turns out to be deregulated affecting adhesion properties and molecule release that finally foster muscle inflammation [29].

Some studies on DMD patients showed a pro-fibrotic and anti-myogenic environment [15,40], but the modifications of skeletal muscle ECM during DMD progression and the upstream molecular mechanisms responsible of muscular fibrosis are not completely known yet.

In this study, we aimed at analysing muscle fibrosis at the functional and morphological level to understand how fibrosis affects different muscles during the progression of the pathology in young and adult mdx mice. Since GAGs and PGs are key components of connective tissue ECM, they were also evaluated. Finally, we investigated in vitro the underlying collagen turnover pathways in cultured primary muscle fibroblasts. For this purpose, in vivo and in vitro evaluations were performed on mdx mouse models that have been extensively used to investigate DMD pathophysiological mechanisms [35].

We first analysed the collagen content in histological sections from QD, TA, GC, and DF muscles of young and adult wt and mdx mice. Collagen content in the endomysium of different muscles of young and adult mice significantly increased in QD and DF. This finding suggests that in adult mice QD and DF are characterised by a more abundant collagen content in the ECM, likely related to an increased functional demand compared to TA and GC.

When we focused on young and adult mdx mice, we observed a significant increase of the fibrosis index in only in DF in 1-month-old mice, while, in 5-month-old mice, it significantly increased also in QD and GC. This finding points to a progression of fibrosis from the onset to the adult age, strengthened by the significant correlation with the percentage of centronucleated muscle fibres and confirming the tight relationship between DMD-associated fibrogenesis and the extent of muscle degeneration/regeneration. These findings are consistent with the timing of mdx pathogenesis that is progressive and involves an increase in blood creatine kinase levels and, histologically, muscle degeneration with necrotic foci together with muscle regeneration [41]. The disease progression is characterised by distinct waves [42]. In mdx mice, an initial phase of extensive degeneration/regeneration of muscle fibres is observed at a young age (2–4 weeks) with marked myofibre necrosis, apoptosis, and inflammation promote regeneration process; this leads to the formation of newly differentiating myofibres with centralised nuclei and an increased heterogeneity in myofibre size. The regeneration process, alternating with degeneration, continues until 12 weeks of age and, at around 20 weeks, muscles become hypertrophic and fibrosis starts, further increasing at later stages [43,44]. The late dystrophic phenotype appears after 15 months and is characterised by muscle wasting, scoliosis, and heart failure [45,46].

Although the severity of fibrosis and loss of function in many mdx mouse muscles is less pronounced than in human patients, thoracic DF, a striated respiratory muscle in constant use, displays degeneration most closely related to the human condition with extensive fibrosis evident from 12 weeks of age [47,48]. This evidence is consistent with our results showing that DF is the most fibrotic muscle in mdx mice at different ages. Interestingly, our data show that DF fibrosis degree within the adult mdx mice group inversely correlated with muscle force scores measured by WBT and with physical performances. A further demonstration of the tight structure–function relationship was recently demonstrated in DF by the electron scanning microscope (SEM) analysis of collagen fibres in the epimysium of mice. SEM analysis revealed that also collagen arrangement is age- and disease-dependent, since during disease progression an increased collagen fibre straightness and alignment were described [49].

BYG is a component of the ground substance of ECM connective tissues. It is a small leucine-rich proteoglycan containing two chondroitin or dermatan sulphate side chains at their N-terminal ends [50]. In addition to operating in a broad biological context, BYG also displays tissue-specific affinities to certain receptors and structural components, thereby playing a crucial role in maintaining skeletal muscle integrity [51]. More in detail, BYG elicits some important functions in muscles, including the binding to collagens and TGF-β (that acts as a key regulator of ECM homeostasis), and interacts with the β-dystroglycan [52], a component of the DGC of the muscle cell membrane that is impaired in DMD. It also binds α- and γ-sarcoglycan [53]. It was previously demonstrated that, in the ECM imbalance associated with DMD, also the synthesis of several proteoglycans increased in mdx mice, including the biglycan (BYG). Its increased expression was reported at the protein level by immunohistochemistry [52] as well as at the molecular level [54]. Increased levels of BYG were also found in skeletal muscle of DMD patients [55,56]. Our results show that BYG expression, mostly localised around and associated with myofibres, strongly increased in DF of adult mdx mice, according to previous reports. Moreover, this finding is consistent with our results obtained from the histological evaluations of muscle structure and fibrosis. In this scenario, BYG could play a role in the assembly of the DGC, with its upregulation potentially supporting mdx muscle regeneration. However, the role of PGs in DMD-associated fibrosis needs to be further investigated.

Interstitial collagen is the main component of ECM of connective tissues, and in skeletal muscles, is located in the endomysium, perimysium, and epimysium. Its content is dependent on a finely tuned turnover acting at the level of collagen synthesis, maturation, and degradation [1].

The key players responsible for ECM remodelling and collagen turnover in skeletal muscle are resident fibroblasts; however, muscle cells are also able to synthesise and secrete numerous connective components and ECM-related molecules, suggesting their active and direct involvement in connective tissue homeostasis [57,58].

To dissect the molecular mechanisms responsible for DMD-related muscle fibrosis, we analysed the overall collagen turnover pathways in primary muscle fibroblasts obtained from QD. Our results showed that COL-I and COL-III levels secreted in the cell culture medium are similar in wt and mdx and are not affected by age.

Newly synthesised collagen undergoes maturation by cross-linking that is an important requirement for providing fibril strength, stabilisation, and alignment. Collagen maturation is based on the formation of covalent cross-links following the hydroxylation of amino acid residues by lysyl oxidase (LOX) and lysyl hydroxylase (LH) [13,14]. Here, we show that LOX was not significantly affected. By contrast, gene expression for LH2b, the isoform mostly involved in fibrogenesis [14], resulted up-regulated in mdx fibroblasts compared to wt and in adult mdx compared to young mdx fibroblasts. This finding is consistent to the hypothesis that, even if collagen secretion was unmodified, the higher cross-linking in mdx fibroblast could act as a key mechanism responsible for collagen accumulation in DMD-associated fibrosis. In fact, a higher level of cross-linking renders collagen less susceptible to its degradation, thus contributing to its accumulation.

Since collagen content is the result of a finely regulated dynamic balance between its synthesis and degradation driven by MMPs, degradation pathways were also investigated. Interstitial collagen breakdown is driven by MMP-1, needed to cleave the intact collagen triple helix, followed by other proteases [59]. Both its activation and activity are regulated in a stoichiometric 1:1 relationship by TIMPs [60]. Our results show that MMP-1 and MMP-2 levels were similar in all the considered experimental groups. By contrast, TIMPs are expressed at higher extent in mdx mice. Indeed, TIMP-1 mRNA levels were more evident in adult mdx mice, and TIMP-2 was strongly up-regulated in adult mdx compared to wt mice. Mechanistically, this finding points to collagen degradation inhibition as a major determinant of muscular fibrosis in adult mdx mice. The pattern of expression of TIMPs is consistent with their contribution to fibrosis activation and maintaining in the adult, when is more evident, rather than during the progression of the pathology.

## 4. Materials and Methods

### 4.1. Animal Model

All procedures involving mice were performed in accordance with the Italian law on animal care (D.L. 26/2014) as well as the European Directive (2010/63/UE) and animal experimentation was approved by the Ministero della Salute (approval no. 978/2017-PR). Wild-type (wt) (C57BL/10ScSnJ) and dystrophic mdx (C57BL/10ScSnDmdmdx/J) mice were housed in an environmentally controlled room (23 ± 1 °C, 50 ± 5% humidity) with a 12 h light/dark cycle and provided food and water ad libitum. Skeletal muscles were obtained from young (1-month-old) (n = 5) and adult (5-months-old) (n = 5) wt and mdx mice and were either processed for histological analysis or digested using collagenase to obtain primary fibroblasts for in vitro molecular analysis.

### 4.2. Functional Tests, Collagen Content, and Fibrosis Analysis

#### 4.2.1. Whole-Body Tension Test

The whole-body tension (WBT) force test was used to determine the ability of mice to exert tension in a forward pulling manoeuvre that is elicited exerted by the forelimb and hindlimb musculature by stroking the tail of the mice. It is thought to reflect the maximal acute phasic force the mouse can achieve to escape a potentially harmful event [61]. The tails were connected to an MP150 System transducer (BIOPAC Systems, Inc., Goleta, CA, USA) and forward pulling movements were elicited by a standardized stroke of the tail, and the corresponding pulling tensions were recorded using the AcqKnowledge software recording system (Version 3.8.2, GenuineIntel, 1992-2006 BIOPAC Systems, Inc., Goleta, CA, USA). Between 20 and 30 strokes of pulling tensions were generally recorded. The WBT was calculated as the average of the top ten or top five performances (WBT 5/WBT 10) normalised on the body weight of mice in grams; it represents the maximum phasic tension that can be developed.

#### 4.2.2. Exhaustion Treadmill Test

Animals were made to run horizontally on the standard treadmill machine Exer 3/6 Treadmill (Columbus Instruments, Columbus, OH, USA) to assess their resistance to fatigue. The exhaustion treadmill test was performed after an appropriate acclimatizing period. The assay consisted of horizontal running for 5 min at 8 cm/s; then, the speed was increased by 2 cm/s each minute until reaching either 50 cm/s or mice exhaustion as reported in the literature [62] and the TREAT-NMD SOP [63]). Exhaustion was defined as the inability of the animal to return to running within 10 s after direct contact on an electric stimulus grid. Running times and distances were calculated by the software. Mice were sacrificed at least 24 h after the exhaustion treadmill test.

#### 4.2.3. Histological Analysis and Histochemistry

Muscle fragments were immediately fixed in 4% formalin in 0.1 M phosphate-buffered saline (PBS), pH 7.4, routinely dehydrated, paraffin-embedded, and serially cut (thickness 5 μm). Sections of quadriceps (QD), tibialis anterior (TA), gastrocnemius (GC), and diaphragm (DF) muscles were stained with freshly made haematoxylin–eosin to evaluate the cell and tissue morphology. Sirius red, Herovici’s stain, and Alcian blue were used to evaluate collagen content, newly deposited collagen, and GAGs/PGs content, respectively.

#### 4.2.4. Sirius Red Staining

For Sirius red staining, to specifically stain fibrillary collagen, slides were deparaffinised and immersed for 30 min in saturated aqueous picric acid containing 0.1% Sirius red F3BA (Sigma-Aldrich, Milan, Italy) [64]. The slides were photographed with a digital camera connected to a Nikon Eclipse 80i microscope. Collagen content was quantified blind by three different operators by image analysis in at least 8 random fields and expressed as fibrosis index, that is, the ratio between the collagen relative to the whole section area analysed, expressed as a %.

On the same sections analysed for collagen content, the number of centronucleated skeletal muscle fibres was quantified in order to analyse the eventual relationship between skeletal muscle injury and fibrosis.

#### 4.2.5. Herovici’s Staining

The Herovici’s stain is a variant of a picropolychrome stain to selectively stain the nuclei, the cytoplasm, and the connective tissue. More in detail, it allows to differentially stain in purple the mature and more cross-linked collagen (mostly type I collagen) and in blue the less mature and young collagen (mostly type III collagen in reticular fibres) [65,66] and can be used to study the ECM remodelling response in a connective tissue.

The slides stained with Herovici were photographed by a digital camera connected to a Nikon Eclipse 80i microscope. Sections for each muscle and each stain were analysed blind by three different operators.

### 4.3. Analysis of Non-Collagen Ground Substance Components

#### 4.3.1. Alcian Blue Staining

Alcian blue was used to obtain specific stain for mucopolysaccharides (GAGs and PGs). For this purpose, sections were stained with Alcian blue in sodium acetate buffer, pH 5.8, containing different MgCl_2_ concentrations in order to selectively stain different mucopolysaccharides. In particular, using 0.025 M MgCl_2_ all the acid mucopolysaccharides are stained in blue; using 0.3 M and 0.65 M MgCl_2_, respectively, sulphated acid mucopolysaccharides and strongly sulphated acid mucopolysaccharides can be observed.

The slides stained with Alcian Blue were photographed by a digital camera connected to a Nikon Eclipse 80i microscope. Sections for each muscle and each MgCl_2_ concentration used were analysed blind by three different operators using a semiquantitative grading scale to assess both the intensity as well as the abundance of the staining in the endomysium. To assess the Alcian blue intensity, the semiquantitative score was based on four-point scoring system, where 0 is very faint staining, 1 faint staining, 2 moderate staining, and 3 strong staining. To assess the distribution and abundance of the Alcian blue staining, the semiquantitative score was based on four-point scoring system, where 0 Alcian blue in less of the 25% of the section, 1 is Alcian blue accumulation in 26–50% of the section, 2 is Alcian blue accumulation in 51–75% of the section, and 3 is Alcian blue accumulation in >75% of the section. The two semiquantitative scores were combined to obtain an index to describe GAG/PG content and expressed as means ± standard deviation (SD).

#### 4.3.2. Biglycan Immunohistochemistry

After the deparaffinisation and rehydration of DF sections, slides were incubated in 3% H_2_O_2_ in PBS for 20 min at 37 °C to block the endogenous peroxidase activity, and with proteinase K in Tris-EDTA buffer (TE), pH 8 for 20 min at 37 °C for antigen retrieval. Sections were incubated with primary antibody goat anti-biglycan (1:200 in PBS, Abcam, Cambridge, UK) overnight (o.n.) at 4 °C. After incubation with horseradish peroxidase (HRP)-conjugated secondary antibody for 1 h at room temperature (dilution 1:400 in PBS), chromogenic 3,3′-diaminobenzidine (DAB) substrate was added to visualize the expression of the target proteins. Negative controls were obtained omitting the primary antibody. The sections were observed under a light microscope (Nikon Eclipse E600, Nikon, Tokyo, Japan) and photographed by a digital camera.

### 4.4. In Vitro Analysis of Muscle Fibroblasts

Primary cell cultures of muscle fibroblasts were obtained from QD muscles. Muscle fragments were carefully washed in sterile PBS and incubated in a solution of Dulbecco’s Modified Eagle Medium (DMEM) and collagenase I 4 mg/mL (Merck Life Sciences, Milan, Italy). Cell suspension was filtered and plated in a T25 flask. Cells were cultured in DMEM containing 2 mM glutamine, antibiotics (100 U/mL penicillin, 0.1 mg/mL streptomycin), and 0.25 μg/mL amphotericin B (Euroclone, Pero, Milan, Italy) at 37 °C in a humidified atmosphere containing 5% CO_2_. Cells were subcultured in T25 flasks and cell viability was determined by Trypan blue staining. For molecular evaluations, confluent fibroblasts were cultured in duplicate in 6-well multi-well plates and analysed at the fifth passage, adding ascorbic acid (200 μM) to DMEM to preserve collagen synthesis, and harvested after 48 h.

#### 4.4.1. Gene Expression Analysis

Muscle fibroblasts were harvested and total RNA isolated (Tri-Reagent, Sigma-Aldrich, Milan, Italy). A total of 1 μg of total RNA was reverse-transcribed in 20 μL final volume of reaction mix (Biorad, Segrate, Milan, Italy). Gene expression for long lysyl hydroxylase 2 (LH2b), lysyl oxidase (LOX), and tissue inhibitor of matrix metalloproteinase 1 and 2 (TIMP-1, TIMP-2) was analysed by real-time RT-PCR in samples run in triplicate. Glyceraldehyde 3-phosphate dehydrogenase (GAPDH) was used as the internal control to normalise the mRNA levels of the target genes in each sample.

The primers sequences were the following: GAPDH: sense CCCTTCATTGACCTCAACTACATG, antisense TGGGATTTCCATTGATGACAAGC; LH2b: sense ACCACGATGCCTCAACCTTT, antisense CGTGCAAATGTGTGAGCCTC; LOX: sense CCCAGCCACATAGATCGCAT, antisense TGGATGTAGTAGGGGTCGGG; TIMP-1: sense TTCTTGGTTCCCTGGCGTAC, antisense GCAAAGTGACGGCTCTGGTA; TIMP-2: sense GCTCCAACCCTGTCCTAACC, antisense GCACAACACGAAAATGCCCT. Each sample was analysed in triplicate in a Bioer LineGene 9600 thermal cycler (Bioer, Hangzhou, China). The cycle threshold (Ct) was determined and gene expression levels relative to that of GAPDH were calculated using the delta CT method.

#### 4.4.2. Slot Blot

Collagen type I and III (COL-I and COL-III) and matrix metalloproteinase (MMP)-1 protein levels secreted by muscle fibroblasts were analysed by Slot Blot in serum-free cell supernatants as previously described [67]. After blocking, membranes were incubated for 1 h at room temperature with primary polyclonal antibodies to COL-I (1:1000 in 1X Tris-Buffered Saline plus 0.1% Tween 20, TBST) (Sigma-Aldrich, Milan, Italy), COL-III (1:1000 in TBST) (Sigma-Aldrich), or MMP-1 (1 μg/mL in TBST) (Millipore, Milan, Italy). After 1 h incubation with HRP-conjugated antibody (1:20,000 in TBST), immunoreactive bands were revealed by the Amplified Opti-4CN substrate (Amplified Opti-4CN, Bio Rad, Segrate, Milan, Italy) and quantified by densitometric scanning (UVBand, Eppendorf, Milan, Italy).

#### 4.4.3. SDS-Zymography

Serum-free cell culture supernatants (5 μg of total protein per sample) were run on 10% polyacrylamide gels co-polymerised with 1 mg/mL type I gelatine. The gels were run at 4 °C and, after Sodium Dodecyl Sulphate—Polyacrylamide gel electrophoresis (SDS-PAGE), they were washed twice in 2.5% Triton X-100 for 30 min each to remove SDS and incubated overnight in a substrate buffer at 37 °C (50 mM Tris-HCl, 5 mM CaCl_2_, 0.02% NaN_3_, pH 7.5). After staining and destaining the gels, MMP gelatinolytic activity was detected as clear bands on a blue background. Clear bands on the zymogram were quantified by densitometric scanning (UVBand, Eppendorf, Milan, Italy).

### 4.5. Statistical Analysis

Statistical analysis was performed by the Prism v 9.3 software (GraphPad Software, San Diego, CA, USA). Data were expressed as mean ± standard deviation (SD). Comparison of groups was calculated using a one-way ANOVA. Differences associated with *p*-values lower than 5% were considered significant.

## 5. Conclusions

Physiological collagen content increased at a different extent in the different skeletal muscles in wt mice. In mdx mice, fibrosis was more evident in QD and DF, suggesting that these are the muscles mainly affected in DMD. Indeed, DF, playing a crucial role in breathing, becomes highly fibrotic also in humans.

In this context, higher collagen cross-linking and MMP inhibition could act as major players in the mechanisms leading to muscular fibrosis, contributing differently to collagen accumulation in the progression of DMD. In young mice, fibrosis seems be dependent on increased collagen cross-linking. Conversely, in adult mice, both increased collagen cross-linking and the inhibition of collagen degradation could act as major molecular mechanisms to further favour the expansion and the persistence of the fibrotic remodelling of dystrophic muscles.

In a context in which no resolutive therapy for DMD has been found yet, fibrosis still constitutes a significant pathological trait to be treated [30,31]. The modulation of scar fibrotic process can slow muscle function degeneration, supporting tendon physiological mobility and pharmacological interventions by enhancing the amount of muscle mass available for therapy.

In conclusion, our results could contribute to the characterisation of the DMD-related fibrosis progression and the underlying mechanisms in order to find new therapeutic targets to effectively prevent dystrophic muscle degeneration.

## Data Availability

The data presented in this study are available upon request from the corresponding author.

## References

[1] Kjaer M. (2004). Role of extracellular matrix in adaptation of tendon and skeletal muscle to mechanical loading. Physiol. Rev..

[2] Gillies A.R., Lieber R.L. (2011). Structure and function of the skeletal muscle extracellular matrix. Muscle Nerve.

[3] Lund D.K., Cornelison D.D. (2013). Enter the matrix: Shape, signal and super-highway. FEBS J..

[4] Avery N.C., Bailey A.J. (2005). Enzymic and non-enzymic cross-linking mecha-nisms in relation to turnover of collagen: Relevance to aging and exercise. Scand. J. Med. Sci. Sports.

[5] Ramaswamy K.S., Palmer M.L., Van Der Meulen J.H., Renoux A., Kostrominova T.Y., Michele D.E., Faulkner J.A. (2011). Lateral transmission of force is impaired in skeletal muscles of dystrophic mice and very old rats. J. Physiol..

[6] De Rezende Pinto W., De Souza P., Oliveira A. (2015). Normal muscle structure, growth, development, and regeneration. Curr. Rev. Musculosklet. Med..

[7] Turrina A., Martinez-Gonzalez M.A., Stecco C. (2013). The muscular force transmission system: Role of the intramuscular connective tissue. J. Bodyw. Mov. Ther..

[8] Campbell K.P., Stull J.T. (2003). Skeletal muscle basement membrane sarcolemma-cytoskeleton interaction minireview series. J. Biol. Chem..

[9] Kovanen V. (2002). Intramuscular extracellular matrix: Complex environment of muscle cells. Exerc. Sport Sci. Rev..

[10] Sanes J.R. (2003). The basement membrane/basal lamina of skeletal muscle. J. Biol. Chem..

[11] Lieber R.L., Ward S.R. (2013). Cellular mechanisms of tissue fibrosis. 4. Structural and functional consequences of skeletal muscle fibrosis. Am. J. Physiol. Cell Physiol..

[12] Mahdy M.A.A. (2019). Skeletal muscle fibrosis: An overview. Cell Tissue Res..

[13] Lucero H.A., Kagan H.M. (2006). Lysyl oxidase: An oxidative enzyme and effector of cell function. Cell Mol. Life Sci..

[14] Walker L.C., Overstreet M.A., Yeowell H.N. (2005). Tissue-specific expression and regulation of the alternatively-spliced forms of lysyl hydroxylase 2 (LH2) in human kidney cells and skin fibroblasts. Matrix Biol..

[15] Thomas K., Engler A.J., Meyer G.A. (2015). Extracellular matrix regulation in the muscle satellite cell niche. Connect. Tissue Res..

[16] Gilbert P.M., Havenstrite K.L., Magnusson K.E.G., Sacco A., Leonardi N.A., Kraft P., Nguyen N.K., Thrun S., Lutolf M.P., Blau H.M. (2010). Substrate elasticity regulates skeletal muscle stem cell self-renewal in culture. Science.

[17] Lehti T.M., Silvennoinen M., Kivelä R., Kainulainen H., Komulainen J. (2006). Effects of streptozotocin-induced diabetes and physical training on gene expression of extracellular matrix proteins in mouse skeletal muscle. Am. J. Physiol. Endocrinol. Metab..

[18] Nuckolls G.H., Kinnett K., Dayanidhi S., Domenighetti A.A., Duong T., Hathout Y., Lawlor M.W., Lee S., Magnusson S.P., McDonald C.M. (2020). Conference report on contractures in musculoskeletal and neurological conditions. Muscle Nerve.

[19] Smith L.R., Pichika R., Meza R.C., Gillies A.R., Baliki M.N., Chambers H.G., Lieber R.L. (2021). Contribution of extracellular matrix components to the stiffness of skeletal muscle contractures in patients with cerebral palsy. Connect. Tissue Res..

[20] Bischoff R., Engel A.G., Franzini-Armstrong C. (1994). Myology.

[21] Kaariainen M., Jarvinen T., Jarvinen M., Rantanen J., Kalimo H. (2000). Relation between myofibers and connective tissue during muscle injury repair. Scand. J. Med. Sci. Sports.

[22] Christov C., Chretien F., Abou-Khalil R., Bassez G., Vallet G., Authier F.J., Bassaglia Y., Shinin V., Tajbakhsh S., Chazaud B. (2007). Muscle satellite cells and endothelial cells: Close neighbors and privileged partners. Mol. Biol. Cell.

[23] Hoffman E.P., Brown Jr R.H., Kunkel L.M. (1987). Dystrophin: The protein product of the Duchenne muscular dystrophy locus. Cell.

[24] Emery A.E. (1991). Population frequencies of inherited neuromuscular diseases—A world survey. Neuromuscul. Disord..

[25] Mah J.K., Korngut L., Dykeman J., Day L., Pringsheim T., Jette N. (2014). A systematic review and meta-analysis on the epidemiology of Duchenne and Becker muscular dystrophy. Neuromuscul. Disord..

[26] Gumerson J.D., Michele D.E. (2011). The dystrophin-glycoprotein complex in the prevention of muscle damage. J. Biomed. Biotechnol..

[27] Emery A.E.H., Muntoni F., Quinlivan R.C.M. (2015). Duchenne Muscular Dystrophy.

[28] Deconinck N., Dan B. (2007). Pathophysiology of duchenne muscular dystrophy: Current hypotheses. Pediatr. Neurol..

[29] Kharraz Y., Guerra J., Pessina P., Serrano A.L., Muñoz-Cánoves P. (2014). Understanding the process of fibrosis in Duchenne muscular dystrophy. Biomed. Res. Int..

[30] Grages S.M., Bell M., Berlau D.J. (2020). New and emerging pharmacotherapy for duchenne muscular dystrophy: A focus on synthetic therapeutics. Expert Opin. Pharmacother..

[31] Dzierlega K., Yokota T. (2020). Optimization of antisense-mediated exon skipping for Duchenne muscular dystrophy. Gene Ther..

[32] Bulfield G. (1984). X chromosome-linked muscular dystrophy. Proc. Natl. Acad. Sci. USA.

[33] McGreevy J.W., Hakim C.H., McIntosh M.A., Duan D. (2015). Animal models of Duchenne muscular dystrophy: From basic mechanisms to gene therapy. Dis. Models Mech..

[34] Partridge T.A. (2013). The mdx mouse model as a surrogate for Duchenne muscular dystrophy. FEBS J..

[35] Potter R.A., Griffin D.A., Heller K.N., Peterson E.L., Clark E.K., Mendell J.R., Rodino-Klapac L.R. (2021). Dose-escalation study of systemically selivered rAAVrh74.MHCK7.micro-dystrophin in the mdx mouse model of Duchenne Muscular Dystrophy. Hum. Gene Ther..

[36] Mann C.J., Perdiguero E., Kharraz Y., Aguilar S., Pessina P., Serrano A.L., Munoz-Canoves P. (2011). Aberrant repair and fibrosis development in skeletal muscle. Skelet. Muscle.

[37] Giovarelli M., Zecchini S., Catarinella G., Moscheni C., Sartori P., Barbieri C., Roux-Biejat P., Napoli A., Vantaggiato C., Cervia D. (2021). Givinostat as metabolic enhancer reverting mitochondrial biogenesis deficit in Duchenne Muscular Dystrophy. Pharmacol. Res..

[38] Allen D.G., Gervasio O.L., Yeung E.W., Whitehead N.P. (2010). Calcium and the damage pathways in muscular dystrophy. Can. J. Physiol. Pharmacol..

[39] Cros D., Harnden P., Pellissier J.F., Serratrice G. (1989). Muscle hypertrophy in duchenne muscular dystrophy. J. Neurol..

[40] Zanotti S., Gibertini S., Mora M. (2010). Altered production of extracellular matrix components by muscle-derived Duchenne muscular dystrophy fibroblasts before and after TGF- beta1 treatment. Cell Tissue Res..

[41] Rodrigues M., Echigoya Y., Maruyama R., Lim K.R., Fukada S.I., Yokota T. (2016). Impaired regenerative capacity and lower revertant fibre expansion in dystrophin-deficient mdx muscles on DBA/2 background. Sci. Rep..

[42] McGeachie J.K., Grounds M.D., Partridge T.A., Morgan J.E. (1993). Age-related changes in replication of myogenic cells in rndx mice: Quantitative autoradiographic studies. J. Neurol. Sci..

[43] Grounds M.D., Torrisi J. (2004). Anti-TNFalpha (Remicade) therapy protects dystrophic skeletal muscle from necrosis. FASEB J..

[44] Messina S., Bitto A., Aguennouz M., Minutoli L., Monici M.C., Altavilla D., Squadrito F., Vita G. (2006). Nuclear factor kappa-B blockade reduces skeletal muscle degeneration and enhances muscle function in Mdx mice. Exp. Neurol..

[45] Lefaucheur J.P., Pastoret C., Sebille A. (1995). Phenotype of dystrophinopathy in old mdx mice. Anat. Rec..

[46] Bostick B., Yue Y., Long C., Marschalk N., Fine D.M., Chen J., Duan D. (2009). Cardiac expression of a mini-dystrophin that normalizes skeletal muscle force only partially restores heart function in aged Mdx mice. Mol. Ther..

[47] Stedman H.H., Sweeney H.L., Shrager J.B., Maguire H.C., Panettieri R.A., Petrof B., Narusawa M., Leferovich J.M., Sladky J.T., Kelly A.M. (1991). The mdx mouse diaphragm reproduces the degenerative changes of Duchenne muscular dystrophy. Nature.

[48] Gosselin L.E., Williams J.E. (2006). Pentoxifylline fails to attenuate fibrosis in dystrophic (mdx) diaphragm muscle. Muscle Nerve.

[49] Sahani R., Wallace C.H., Jones B.K., Blemker S.S. (2022). Diaphragm muscle fibrosis involves changes in collagen organization with mechanical implications in Duchenne muscular dystrophy. J. Appl. Physiol..

[50] Krusius T., Ruoslahti E. (1986). Primary structure of an extracellular matrix proteoglycan core protein deduced from cloned cDNA. Proc. Natl. Acad. Sci. USA.

[51] Nastase M.V., Young M.F., Schaefer L. (2012). Biglycan: A multivalent proteoglycan providing structure and signals. J. Histochem. Cytochem..

[52] Bowe M.A., Mendis D.B., Fallon J.R. (2000). The small leucine-rich repeat proteoglycan biglycan binds to alpha-dystroglycan and is upregulated in dystrophic muscle. J. Cell Biol..

[53] Rafii M.S., Hagiwara H., Mercado M.L., Seo N.S., Xu T., Dugan T., Owens R.T., Hook M., McQuillan D.J., Young M.F. (2006). Biglycan binds to alpha- and gamma-sarcoglycan and regulates their expression during development. J. Cell Physiol..

[54] Haslett J.N., Sanoudou D., Kho A.T., Bennett R.R., Greenberg S.A., Kohane I.S., Beggs A.H., Kunkel L.M. (2002). Gene expression comparison of biopsies from Duchenne muscular dystrophy (DMD) and normal skeletal muscle. Proc. Natl. Acad. Sci. USA.

[55] Zanotti S., Negri T., Cappelletti C., Bernasconi P., Canioni E., Di Blasi C., Pegoraro E., Angelini C., Ciscato P., Prelle A. (2005). Decorin and biglycan expression is differentially altered in several muscular dystrophies. Brain.

[56] Fadic R., Mezzano V., Alvarez K., Cabrera D., Holmgren J., Brandan E. (2006). Increase in decorin and biglycan in Duchenne Muscular Dystrophy: Role of fibroblasts as cell source of these proteoglycans in the disease. J. Cell Mol. Med..

[57] Zhang W., Liu Y., Zhang H. (2021). Extracellular matrix: An important regulator of cell functions and skeletal muscle development. Cell Biosci..

[58] Fry C.S., Kirby T.J., Kosmac K., McCarthy J.J., Peterson C.A. (2017). Myogenic progenitor cells control extracellular matrix production by fibroblasts during skeletal muscle hypertrophy. Cell Stem Cell.

[59] Sakai T., Gross J. (1967). Some properties of the products of reaction of tadpole collagenase with collagen. Biochemistry.

[60] Murphy G., Willenbrock F., Crabbe T., O’Shea M., Ward R., Atkinson S., O’Connell J., Docherty A. (1994). Regulation of matrix metalloproteinase activity. Ann. N. Y. Acad. Sci..

[61] DMD_M.2.2.006 Whole Body Tension Measurements. http://www.treat-nmd.eu/downloads/file/sops/dmd/MDX/dmd_m.2.2.006.pdf.

[62] Burdi R., Rolland J.F., Fraysse B., Litvinova K., Cozzoli A., Giannuzzi V., Liantonio A., Camerino G.M., Sblendorio V., Capogrosso R.F. (2009). Multiple pathological events in exercised dystrophic mdx mice are targeted by pentoxifylline: Outcome of a large array of in vivo and ex vivo tests. J. Appl. Physiol..

[63] DMD_M.2.1.003 Use of Treadmill and Wheel Exercise to Assess Dystrophic State. https://treat-nmd.org/wp-content/uploads/2016/08/MDX-DMD_M.2.1.003-34.pdf.

[64] Junqueira L.C., Bignolas G., Brentani R.R. (1979). Picrosirius staining plus polarization microscopy, a specific method for collagen detection in tissue sections. Histochem. J..

[65] Herovici C. (1963). Picropolychrome: Histological staining technic intended for the study of normal and pathological connective tissue. Rev. Fr. D’etudes Clin. Biol..

[66] Lillie R.D., Tracy R.E., Pizzolato P., Donaldson P.T., Reynolds C. (1980). Differential staining of collagen types in paraffin sections: A color change in degraded forms. Virchows Arch. A Pathol. Anat. Histol..

[67] Randelli F., Menon A., Giai Via A., Mazzoleni M.G., Sciancalepore F., Brioschi M., Gagliano N. (2018). Effect of a Collagen-Based Compound on Morpho-Functional Properties of Cultured Human Tenocytes. Cells.

